# The effects of superimposed tilt and lower body negative pressure on anterior and posterior cerebral circulations

**DOI:** 10.14814/phy2.12957

**Published:** 2016-09-15

**Authors:** Michael M. Tymko, Caroline A. Rickards, Rachel J. Skow, Nathan C. Ingram‐Cotton, Michael K. Howatt, Trevor A. Day

**Affiliations:** ^1^ Centre for Heart Lung and Vascular Health School of Health and Exercise Science University of British Columbia Kelowna Canada; ^2^ Department of Biology Faculty of Science and Technology Mount Royal University Calgary Alberta Canada; ^3^ Institute for Cardiovascular & Metabolic Diseases University of North Texas Health Science Centre Fort Worth Texas; ^4^ Faculty of Physical Education and Recreation University of Alberta Edmonton Alberta Canada

**Keywords:** Central hypovolemia, cerebral blood velocity, head‐down tilt, head‐up tilt, lower body negative pressure

## Abstract

Steady‐state tilt has no effect on cerebrovascular reactivity to increases in the partial pressure of end‐tidal carbon dioxide (P_ET_CO_2_). However, the anterior and posterior cerebral circulations may respond differently to a variety of stimuli that alter central blood volume, including lower body negative pressure (LBNP). Little is known about the superimposed effects of head‐up tilt (HUT; decreased central blood volume and intracranial pressure) and head‐down tilt (HDT; increased central blood volume and intracranial pressure), and LBNP on cerebral blood flow (CBF) responses. We hypothesized that (a) cerebral blood velocity (CBV; an index of CBF) responses during LBNP would not change with HUT and HDT, and (b) CBV in the anterior cerebral circulation would decrease to a greater extent compared to posterior CBV during LBNP when controlling P_ET_CO_2_. In 13 male participants, we measured CBV in the anterior (middle cerebral artery, MCAv) and posterior (posterior cerebral artery, PCAv) cerebral circulations using transcranial Doppler ultrasound during LBNP stress (−50 mmHg) in three body positions (45°HUT, supine, 45°HDT). P_ET_CO_2_ was measured continuously and maintained at constant levels during LBNP through coached breathing. Our main findings were that (a) steady‐state tilt had no effect on CBV responses during LBNP in both the MCA (*P* = 0.077) and PCA (*P* = 0.583), and (b) despite controlling for P_ET_CO_2_, both the MCAv and PCAv decreased by the same magnitude during LBNP in HUT (*P* = 0.348), supine (*P* = 0.694), and HDT (*P* = 0.407). Here, we demonstrate that there are no differences in anterior and posterior circulations in response to LBNP in different body positions.

## Introduction

Both lower body negative pressure (LBNP) and changes in body position induce variations in central blood volume (Cooke et al. 2004) and intracranial pressure (Macias et al. 2015; Rosner and Coley 1986; Schwarz et al. 2002). Specifically, LBNP and head‐up tilt (HUT) result in a decrease in both central blood volume and intracranial pressure, while head‐down tilt (HDT) increases both central blood volume and intracranial pressure. Effective physiological compensation to these stressors requires rapid integration of many reflex mechanisms, particularly the cardiovagal and vascular sympathetic baroreflexes. These reflexes are critical for rapid regulation of heart rate and vascular resistance in humans in order to maintain mean arterial pressure (MAP), and subsequently, cerebral perfusion pressure. Recent studies have demonstrated that changes in MAP associated with steady‐state tilt and lower body positive pressure (90°HUT; 90°HDT), are within the autoregulatory capacity of the brain, and thus have no effect on steady‐state CBF (Gelinas et al. 2012; Perry et al. 2013), nor on cerebrovascular reactivity to increasing and decreasing partial pressure of arterial CO_2_ (PaCO_2_) (Tymko et al. 2015), likely due to compensatory changes in intracranial pressure, and thus cerebral perfusion pressure (Macias et al. 2015; Tymko et al. 2015). To date, however, only one study compared regional CBF regulation during LBNP stress in supine and HUT (Deegan et al. 2010), and none during HDT positions when central blood volume, intracranial pressure, and MAP are likely elevated. This might be attributed to the difficulty in developing an LBNP apparatus that can accommodate HUT and HDT body positions.

Accumulating evidence supports the notion of differentially regulated blood flow in the anterior and posterior cerebral circulations. For example, differences in regional CBF regulation has been demonstrated in response to changes in PaCO_2_ (Sato et al. 2012b; Skow et al. 2013; Willie et al. 2012), hyperthermia (Bain et al. 2013), administration of indomethacin (Hoiland et al. 2015), and during changes in central blood volume such as during tilt (Tymko et al. 2015), thigh cuff release (Sato et al. 2012a), and LBNP (Kay and Rickards 2016; Lewis et al. 2015; Ogoh et al. 2015). However, there is little agreement within the available literature of regional CBF regulation (i.e., anterior vs. posterior cerebral circulation comparisons), especially in terms of orthostatic stress. For example, during reductions in central blood volume (i.e., steady‐state tilt, LBNP, or thigh cuff release), studies have proposed that the anterior cerebral circulation is more responsive compared to the posterior circulation (Kay and Rickards 2016; Ogoh et al. 2015; Sato et al. 2012a; Tymko et al. 2015), and vice‐versa (Lewis et al. 2015), and other studies have suggested that the anterior and posterior cerebral circulation respond in the same fashion (Deegan et al. 2010; Lewis et al. 2014). Collectively, the current evidence on regional differences in cerebral blood flow suggests that the posterior cerebral circulation is more capable of maintaining a constant supply of blood flow compared to the anterior cerebral circulation during central hypovolemia (Kay and Rickards 2016; Ogoh et al. 2015; Sato et al. 2012a; Tymko et al. 2015). Reasoning for this is unclear, however, from an evolutionary standpoint, it makes sense that the posterior cerebral circulation be better fit for maintaining blood flow since it supplies blood to brain regions responsible for crucial homoeostatic functions such as the medulla oblongata, cerebellum, hypothalamus, thalamus, and brainstem (Tatu et al. 1996).

A physiological response that commonly occurs during LBNP is involuntary hyperventilation, resulting in a reduction in PaCO_2_. Blood flow through the cerebrovasculature is highly sensitive to changes in PaCO_2_. During hypercapnia (high PaCO_2_), blood flow through the brain vasculature increases due to downstream vasodilatation of arterioles, while during hypocapnia (low PaCO_2_), blood flow through the brain vasculature decreases due to arteriolar vasoconstriction (Kety and Schmidt 1948; Willie et al. 2014; Wolff et al. 1930). This physiological response aids in the tight regulation of substrate delivery, metabolic waste wash‐out, and acid–base balance, which is particularly important for breathing stability (Ainslie and Duffin 2009). However, changes in PaCO_2_ can be a potential confound when quantifying the CBF responses to physiological perturbations such as orthostatic stress, as baroreflex‐associated changes in ventilation can acutely alter PaCO_2_, and thus CBF (Brunner et al. 1982; Heymans and Bouckaert 1930). Although some recent work has controlled P_ET_CO_2_, a surrogate of PaCO_2_, while investigating CBF responses to tilt‐table testing and LBNP (Gelinas et al. 2012; Lewis et al. 2014, 2015), it is surprising that relatively few studies investigating cerebrovascular responses to LBNP have instituted PaCO_2_ control (Brown et al. 2003; Cencetti et al. 1997; Levine et al. 1994; Ogoh et al. 2015). In this study, we attempted to control this PaCO_2_ confounding variable.

The primary purpose of this study was to investigate the regional cerebrovascular responses to simultaneous changes in body position and LBNP stress. Although it is well established that body position results in changes in central blood volume and intracranial pressure (e.g., both decrease with HUT and increase with HDT) (Cooke et al. 2004; Macias et al. 2015; Rosner and Coley 1986), less is known about whether body position has an effect on cerebrovascular regulation during LBNP, particularly in the position of HDT where central blood volume and intracranial pressure are increased. Additionally, it is currently unknown whether alterations in intracranial pressure can affect the cerebral blood flow response to LBNP‐induced central hypovolemia, furthermore, the differences between the anterior and posterior circulations under these conditions remains unclear (Deegan et al. 2010; Kay and Rickards 2016; Lewis et al. 2014, 2015; Ogoh et al. 2015). Using a novel, purpose‐built experimental apparatus, we tested the hypotheses that (a) anterior and posterior cerebral blood velocity responses during lower body negative pressure would not be altered with head‐up tilt and head‐down tilt, and (b) cerebral blood velocity in the anterior cerebral circulation would decrease to a greater extent compared to the posterior cerebral circulation during lower body negative pressure, when controlling for the partial pressure of arterial carbon dioxide (via end‐tidal carbon dioxide).

## Materials and Methods

### Ethical approval

All experimental procedures and protocols were reviewed and approved by the Mount Royal University Human Research Ethics Board (Ethics ID 2011‐91Sa) and conformed to the Declaration of Helsinki and the Canadian Government Tri‐Council Policy Statement on research ethics (TCPS2). All participants provided written informed consent prior to participation in this study.

### Participants

All experiments were conducted at Mount Royal University (Calgary, AB). Recruited participants (*n* = 14) were required to complete a health history questionnaire to ensure normal pulmonary, cardiovascular, and cerebrovascular health. Participants were male, between the ages of 18–40 years, had a body mass index <30 kg/m^2^, and were normotensive (systolic blood pressure 123.0 ± 3.2, diastolic blood pressure 60.8 ± 3.6). Female participants were excluded due to difficulty creating an airtight seal due to the large size of the LBNP kayak skirt (see below). Participants were also nonsmokers, had no reported previous history of respiratory, cardiovascular, cerebrovascular diseases, and were not taking any medications. Participants were asked to refrain from vigorous physical activity, alcohol consumption, and caffeine for at least 12 h prior to experimentation.

### Instrumentation

**Figure 1:**
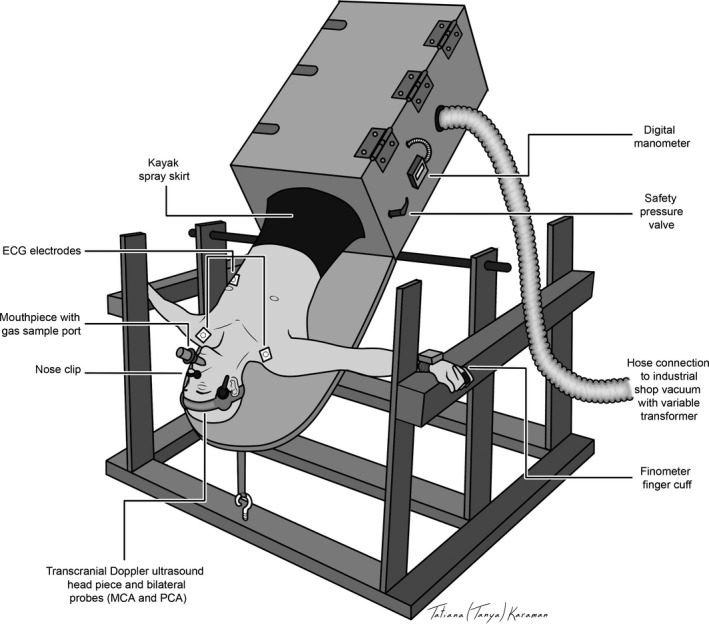
Schematic of a participant positioned in 45°HDT in the custom‐built integrated tilt and LBNP apparatus. The participant was instrumented with a mouthpiece and nose clip (to sample P_ET_CO_2_), Finometer (for monitoring beat‐by‐beat blood pressure), ECG leads (for monitoring HR), and TCD (for monitoring MCAv and PCAv). The LBNP chamber was secured to a custom‐built frame on an axle, and was connected to an industrial shop‐vacuum to generate negative pressure, and a digital manometer was used to monitor pressure within the chamber. The participant was secured inside the chamber with ankle restraints, and they were sealed within the chamber by means of a kayak spray skirt. ECG, electrocardiogram; HR, heart rate; LBNP, lower body negative pressure; PCA, posterior cerebral artery.

Upon arrival on the experimental day, participants placed a kayak skirt, which was integrated to the lid of the LBNP chamber, around their waist and positioned themselves within the LBNP chamber in the upright (i.e., 90°HUT) position. The kayak skirt was secured to the inside of the lid of the LBNP chamber (see Fig. 1). The LBNP chamber was then moved into the supine position, where it was confirmed that the participant had the kayak skirt positioned at the level of the iliac crest, and a stretchable waist belt was wrapped around the kayak skirt to ensure a tight seal of the LBNP chamber. The LBNP vacuum was turned on briefly to −50 mmHg in order to familiarize the participant with the LBNP stress, and to ensure that adequate LBNP could be achieved. The moderate level of LBNP was chosen (i.e., −50 mmHg) in order to elicit a cardiovascular response in each body position, while allowing all participants to withstand at least 5 min of LBNP in each body position (particularly in HUT).

### Respiratory and cardiovascular measures

All respiratory and cardiovascular measurements were collected at 200 Hz using an analog‐to‐digital converter (Powerlab/16SP ML880; ADInstruments; Colorado Springs, CO) and analyzed offline using commercially available software (ADI LabChart Pro 7.2; ADInstruments, Colorado Springs, CO). Participants breathed through a mouthpiece (with nose clip), where expired CO_2_ was sampled and measured in percent (ADI ML206; ADInstruments). The gas analyzer was calibrated with known gas concentrations prior to each test. The partial pressure of end‐tidal CO_2_ (P_ET_CO_2_; mmHg) was calculated using peak analysis and corrected for body temperature and pressure saturated (BTPS) using the daily atmospheric pressure.

Participants were instrumented with electrocardiogram (ECG) electrodes in lead II configuration in conjunction with a bioamp (ADI ML132; ADInstruments) to derive instantaneous heart rate from the R‐R interval of the ECG. Beat‐by‐beat arterial blood pressure, cardiac output (CO), and stroke volume (SV), were measured using finger photoplethysmography (Finometer Pro, Finapres Medical Systems, Amsterdam, NL). Prior to baseline data collection, the Finometer was calibrated using the return‐to‐flow function, and blood pressure accuracy was confirmed with manual sphygmomanometer. Mean arterial pressure (MAP) was calculated using the area under the curve of the arterial pressure envelope tracing.

### Intracranial blood velocity

Cerebral blood velocity (CBV) in the right middle cerebral artery, (MCA) and left posterior cerebral artery, (PCA) were measured using a 2‐MHz pulsed TCD ultrasound system (PMD150B, Spencer Technologies, Redmond, WA) using insonation techniques described in Willie et al. (2011), and identical to previous studies in our laboratory (Skow et al. 2013; Tymko et al. 2015). The same experienced sonographers (MMT and TAD) insonated and confirmed the MCA and PCA for all participants. Mean CBV was calculated from the envelope of the velocity tracings for both MCA and PCA. Cerebrovascular resistance (CVR) was calculated by dividing MAP by mean CBV.

### Integrated tilt‐table LBNP chamber

Orthostatic stress was elicited using a custom‐built integrated tilt‐table LBNP apparatus (designed and built by the first author, MMT; see Fig. 1). Participants were placed in a rectangular LBNP chamber fastened onto a custom‐built tilt‐table that was suspended on a steel frame by an axle. Ankles were secured using restraints (Teeter Hang‐ups, Tacoma, WA) that were attached to a bar installed inside the LBNP chamber. This LBNP apparatus has the unique ability to place participants in varying body positions, from 90°HUT to 90°HDT. The LBNP chamber was sealed at the participant's iliac crest by use of a kayak spray skirt secured to the LBNP chamber. Pressure across the wall of the box was generated using an industrial 6‐horsepower vacuum, and measured using a digital manometer (DigiMano 1000, 200–200IN, Netech Corporation, Farmingdale, NY). The magnitude of negative pressure was manipulated using a 120‐volt input/140‐volt output variable transformer (Powerstat transformer, The Superior Electric Co, Bristol, CT).

### Experimental protocol

To ensure that experimentally induced changes in blood volume distribution were representative of a true change from baseline, participants were instructed to lie motionless and breathe normally for 20 min before LBNP was applied in each body position (HDT, supine, HUT) (Goswami et al. 2008; Levine et al. 1994). The protocol consisted of an initial 5‐min quiet baseline period in the supine position, after which the pressure inside the LBNP chamber was immediately lowered to −50 mmHg for 10 min or until (a) the participant voluntarily terminated the test due to the onset of subjective symptoms (e.g., tunnel vision, nausea, dizziness, or discomfort) or (b) the participant reached presyncope, which was identified in real time by the investigators by rapid onset of bradycardia, and/or a 30% reduction of systolic blood pressure from baseline values.

To ensure that any changes in CBV were not due to the effects of changing PaCO_2_ (Brown et al. 2003; Gelinas et al. 2012), the investigators coached each participant to breathe at their resting ventilatory rate and depth in order to maintain P_ET_CO_2_ levels at baseline values during LBNP in each position. Immediately after LBNP termination, the participant completed a 15‐min recovery period, and then repeated the same protocol (20‐min baseline, LBNP, and recovery period) in the remaining two randomized tilt positions (45°HDT or 45°HUT). Experimentation in the supine position was always conducted first.

### Data analysis

For each protocol, baseline measurements were averaged over 2 min immediately prior to the start of the LBNP. As participants reached presyncope (defined above) at different time points (notably in 45°HUT, *n* = 8), data within each body position trial were normalized within individual by taking a 30 sec average of all outcome variables immediately prior to 20%, 40%, 60%, 80%, and 100% of the total LBNP protocol duration for that participant; this approach has been used previously (Cooke et al. 2009; Levine et al. 1994; Soller et al. 2008).

To compare differences in respiratory, cardiovascular, and cerebrovascular responses to the LBNP, two‐factor (Factor A: body position, three levels; Factor B: % of LBNP protocol, five levels) repeated measures analysis of variances (RM ANOVAs) were used. To compare data between the MCA and PCA (absolute and relative data), a two‐factor (Factor A: vessel, two levels; Factor B: % of LBNP protocol, five levels) RM ANOVA was used. To compare the differences in protocol time between body positions, a one‐way RM ANOVA was used. Participants who did not reach presyncope during LBNP were designated a 100% LBNP value of 600 sec (10 min). When significant F‐ratios were detected, post hoc comparisons were made using Tukey's post hoc test for pair‐wise comparisons. All statistical analyses were performed using SigmaStat V11.5 (Systat, Chicago, IL). All data in figures are expressed as mean values ± SEM. Statistical significance was defined with a critical *α* < 0.05.

## Results

### Participants

Fourteen participants were recruited for this study, but one participant was excluded from data analysis due to extreme intolerance to LBNP. Thirteen males (mean ± SEM) aged 24.2 ± 1.5 years were subsequently included in the data analysis (height, 178.5 ± 0.7 cm; weight, 84.6 ± 2.7 kg; BMI, 26.5 ± 0.8 kg/m^2^. The MCA was insonated at mean depth of 52.4 ± 1.4 mm and the PCA at a mean depth of 62.4 ± 1.5 mm.

During LBNP, some participants reached presyncope with application of LBNP in the supine position (*n* = 3) and in the 45°HUT position (*n* = 8). In contrast, no participants included in the mean data analysis reached presyncope in the 45°HDT position. The average protocol time for all participants in the 45°HUT position was 467.5 ± 39.5 sec, which was shorter than the protocol time in supine position (565.5 ± 23.1 sec; *P* = 0.014), and 45°HDT (600 ± 0 sec; *P* < 0.001). There was no statistical difference in protocol time between supine and 45°HDT (*P* = 0.534).

### Baseline cardiovascular, respiratory, and cerebrovascular data

Cardiovascular responses during baseline are presented in Table 1. At baseline, SV was lower during 45°HUT by 13.9 ± 3.8% and 13.3 ± 4.7% compared to supine (*P* = 0.001) and 45°HDT (*P* = 0.006) positions, respectively. To compensate for these differences in SV, heart rate (HR) was higher in the 45°HUT position by 11.1 ± 1.6% and 14.8 ± 2.3% compared to supine (*P* = 0.016) and 45°HDT (*P* = 0.001), respectively, and there was subsequently no difference in CO across body positions (*P* = 0.294). MAP was higher during 45°HDT by 10.0 ± 3.7% compared to supine (*P* = 0.003), but there were no differences in MAP between supine and 45°HUT (*P* = 0.370), and 45°HUT and 45°HDT (*P* = 0.089).

**Table 1:** Cardiovascular data during baseline and LBNP in each body position

	Baseline	20%	40%	60%	80%	100%
HR (bpm)						
45°HUT	73.3 ± 2.9^c^^,^ b	87.4 ± 3.3^a^^,^ c ^,^ b	97.3 ± 3.5^a^^,^ c ^,^ b	103.5 ± 3.4^a^^,^ c ^,^ b	107.9 ± 0.3^a^^,^ c ^,^ b	106.8 ± 4.8^a^^,^ c ^,^ b
Supine	65.0 ± 2.3	74.6 ± 2.7^a^	80.3 ± 2.2^a^	80.6 ± 2.5^a^	82.9 ± 1.9^a^^,^ d	82.2 ± 3.6^a^^,^ d
45°HDT	62.2 ± 2.5	73.7 ± 2.0^a^	74.7 ± 2.0^a^	74.4 ± 2.1^a^	73.0 ± 2.2^a^	74.1 ± 2.4^a^
Body position: *P < *0.001; % Protocol: *P < *0.001*;* Interaction: *P < *0.001						
SV (mL)						
45°HUT	96.8 ± 6.1	69.0 ± 3.7^a^	68.1 ± 2.2^a^	63.3 ± 4.0^a^	62.8 ± 4.0^a^	58.2 ± 4.1^a^
Supine	108.1 ± 4.5^b^	76.6 ± 5.3^a^^,^ b	72.8 ± 3.0^a^^,^ b	73.5 ± 3.2^a^^,^ b	71.5 ± 3.8^a^^,^ b	68.9 ± 3.3^a^^,^ b
45°HDT	106.5 ± 3.3^d^	70.5 ± 2.5^a^	73.3 ± 2.6^a^^,^ c	73.4 ± 2.7^a^^,^ c	75.6 ± 3.1^a^^,^ c	75.0 ± 2.2^a^^,^ c
Body position: *P < *0.001*;* % Protocol: *P < *0.001*;* Interaction: *P = *0.001						
CO(L/min)						
45°HUT	6.9 ± 0.3	5.9 ± 0.3^a^^,^ c	6.1 ± 0.3^a^^,^ c	6.4 ± 0.3^a^^,^ c	6.6 ± 0.3^a^^,^ c	5.9 ± 0.3^a^^,^ c
Supine	6.9 ± 0.3	5.6 ± 0.3^a^	5.8 ± 0.2^a^	5.9 ± 0.2^a^	5.9 ± 0.3^a^	5.6 ± 0.3^a^
45°HDT	6.6 ± 0.3	5.2 ± 0.2^a^	5.5 ± 0.3^a^	5.4 ± 0.3^a^	5.5 ± 0.3^a^	5.6 ± 0.3^a^
Body position: *P* < 0.001; % Protocol: *P* < 0.001; Interaction: *P* = 0.059						
MAP (mmHg)						
45°HUT	82.2 ± 3.3	81.7 ± 3.2	82.2 ± 2.4	82.2 ± 2.4	79.9 ± 2.5	73.4 ± 4.1^a^
Supine	79.0 ± 3.8	79.2 ± 2.5	80.8 ± 2.6	80.8 ± 2.3	79.5 ± 2.3	75.1 ± 4.5
45°HDT	87.4 ± 2.3^d^	82.8 ± 1.8	84.3 ± 1.8	85.3 ± 1.9	84.6 ± 1.8	86.8 ± 1.8^d^^,^ b
Body position: *P* = 0.009; % Protocol: *P* = 0.008; Interaction: *P* < 0.001.						

*P*‐values for main effects and interactions are displayed underneath each variable.

HR, heart rate; SV, stroke volume; CO, cardiac output; MAP, mean arterial pressure; HUT, head‐up tilt.

a *P* < 0.05, versus baseline.

b *P* < 0.05, 45° HUT versus supine.

c *P* < 0.05, 45°HUT versus 45°HDT.

d *P* < 0.05, Supine versus 45°HDT.

Cerebrovascular and respiratory responses during baseline are presented in Table 2. No differences were found in MCAv (*P* = 0.055), PCAv (*P* = 0.676), or PCAv CVR (*P* = 0.118), across all body positions at baseline. However, MCAv CVR was greater in 45°HUT compared to supine (*P* = 0.017), but there was no difference detected between 45°HUT and 45°HDT (*P* = 0.860) nor between supine and 45°HDT (*P* = 0.052). No differences were found in baseline P_ET_CO_2_ between body positions (*P* = 0.757).

**Table 2:** Cerebrovascular and respiratory data during baseline and LBNP in each body position

	Baseline	20%	40%	60%	80%	100%
MCAv (cm/sec)						
45°HUT	55.2 ± 3.0	51.2 ± 3.1^a^	50.8 ± 3.0^a^	49.9 ± 2.6^a^	48.5 ± 3.0^a^	45.2 ± 3.2^a^
Supine	59.8 ± 2.0	54.4 ± 2.0^a^	55.4 ± 2.6^a^	55.8 ± 2.3^a^	52.6 ± 2.1^a^	49.9 ± 1.6^a^
45°HDT	59.3 ± 2.3	54.3 ± 2.8^a^	54.6 ± 3.0^a^	54.3 ± 2.4^a^	54.1 ± 2.9^a^	53.6 ± 2.6^a^
Body Position: *P* = 0.077; % Protocol: *P* < 0.001; Interaction: *P* = 0.053						
MCAv CVR (mmHg/cm/sec)						
45°HUT	1.55 ± 0.10^b^	1.67 ± 0.11^b^^,^ a	1.79 ± 0.11^b^^,^ a	1.70 ± 0.10^b^^,^ a	1.72 ± 0.10^b^^,^ a	1.69 ± 0.11^b^^,^ a
Supine	1.36 ± 0.09	1.50 ± 0.09^a^	1.49 ± 0.08^a^	1.50 ± 0.09^a^	1.56 ± 0.10^a^	1.52 ± 0.10^a^
45°HDT	1.51 ± 0.08	1.60 ± 0.12^a^	1.61 ± 0.11^a^	1.62 ± 0.10^a^	1.62 ± 0.09^a^	1.65 ± 0.08^a^
Body Position: *P* = 0.040; % Protocol: *P* < 0.001; Interaction: *P* = 0.970						
PCAv (cm/sec)						
45°HUT	31.7 ± 2.9	29.7 ± 2.9	29.8 ± 3.0	29.4 ± 2.7^a^	28.5 ± 2.7^a^	26.3 ± 2.5^a^
Supine	32.9 ± 2.1	29.8 ± 1.9^a^	30.5 ± 2.0^a^	30.6 ± 2.2^a^	29.2 ± 2.0^a^	27.9 ± 1.9^a^
45°HDT	32.6 ± 2.4	30.5 ± 2.4	29.9 ± 2.3^a^	30.0 ± 2.2^a^	30.0 ± 2.2^a^	30.0 ± 2.2^a^^,^ c
Body Position: *P* = 0.583; % Protocol: *P* < 0.001; Interaction: *P* = 0.046						
PCAv CVR (mmHg/cm/sec)						
45°HUT	2.99 ± 0.28	3.12 ± 0.33^a^	3.16 ± 0.32^a^	3.13 ± 0.30^a^	3.13 ± 0.29^a^	3.09 ± 0.31^a^
Supine	2.59 ± 0.24	2.84 ± 0.24^a^	2.85 ± 0.25^a^	2.87 ± 0.25^a^	2.92 ± 0.24^a^	2.85 ± 0.24^a^
45°HDT	2.92 ± 0.25	2.98 ± 0.27^a^	3.08 ± 0.26^a^	3.07 ± 0.25^a^	3.04 ± 0.23^a^	3.12 ± 0.24^a^
Body Position: *P* = 0.183; % Protocol: *P* < 0.001; Interaction: *P* < 0.542						
P_ET_CO_2_ (mmHg)						
45°HUT	31.5 ± 0.9	30.3 ± 0.7	30.1 ± 0.9	29.9 ± 1.0^a^	29.2 ± 1.0^a^	28.1 ± 0.9^a^
Supine	31.8 ± 1.1	30.0 ± 1.1^a^	30.2 ± 1.1	30.2 ± 1.0	30.3 ± 1.1	29.7 ± 1.2^a^^,^ b
45°HDT	31.6 ± 0.9	30.4 ± 1.1	30.1 ± 1.2	30.9 ± 1.0	30.9 ± 1.0^c^	30.3 ± 1.2^c^
Body Position: *P = *0.219; % Protocol: *P <* 0.001; Interaction: *P < *0.034						

MCAv, middle cerebral artery velocity; MCAv CVR, middle cerebral artery cerebrovascular resistance; PCAv, posterior cerebral artery velocity; PCA CVR, posterior cerebral artery cerebrovascular resistance, P_ET_CO_2_, partial pressure of end‐tidal carbon dioxide, HUT, head‐up tilt; LBNP, lower body negative pressure; PCA, posterior cerebral artery.

a *P* < 0.05, versus baseline.

b *P* < 0.05, 45°HUT versus supine.

c *P* < 0.05, 45°HUT versus 45°HDT.

*P* < 0.05, Supine versus 45°HDT. *P*‐values for main effects and interactions are displayed underneath each variable.

### Effects of body position on cardiovascular variables during LBNP

Table 1 illustrates cardiovascular responses during LBNP in each body position. In all body positions, HR was higher throughout the LBNP protocol compared to baseline (*P* < 0.05). In the 45°HUT position, HR was greater compared to supine and 45°HDT throughout the entire LBNP protocol (*P* < 0.05). In the supine position, HR was greater compared to 45°HDT position at the 80% (*P* = 0.004) and 100% (*P* = 0.021) of the LBNP protocol. As expected, SV was lower during LBNP in all body positions compared to baseline (*P* < 0.05). SV in the supine and 45°HDT position was greater compared to 45°HUT throughout the entire LBNP protocol (*P* < 0.05), with the exception of the 20% LBNP stage, where no difference existed between 45°HUT and 45°HDT (*P* = 0.876). Similar to SV, CO was lower during LBNP in all body positions compared to baseline (*P* < 0.05), but CO was greater in the 45°HUT position compared to 45°HDT (main effect, *P* < 0.001). MAP did not change from baseline nor between body positions at any level of LBNP (*P* > 0.05), except at 100% of the LBNP protocol where MAP was lower compared to baseline in the 45°HUT position (*P* < 0.001), which was likely related to the large number of participants that reached presyncope in 45°HUT (see above). Also, at 100% of LBNP, MAP was higher in the 45°HDT position compared to supine (*P* < 0.001) and 45°HUT (*P* < 0.001).

### Effects of body position on cerebrovascular responses during LBNP

**Figure 2:**
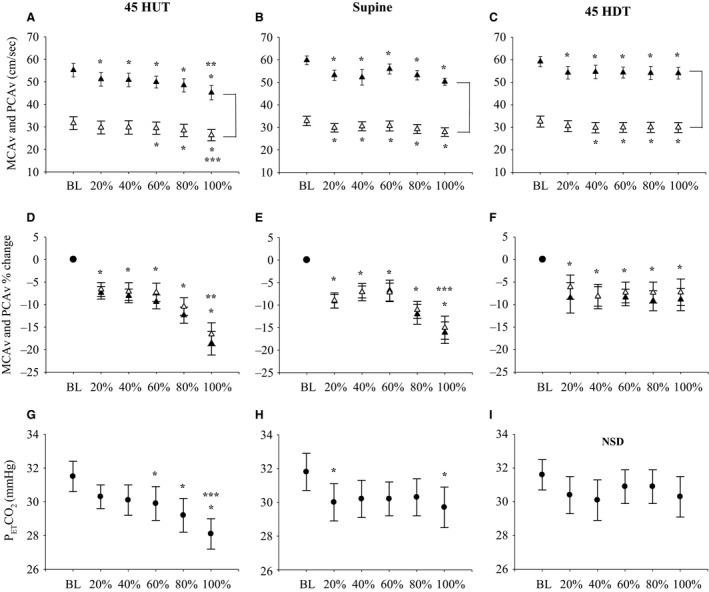
Absolute and relative MCAv and PCAv responses to LBNP in each body position. (▲) represents MCAv data, (∆) represents PCAv data, (•) represents P_ET_CO_2_. Absolute MCAv and PCAv during baseline (BL) and at 20%, 40%, 60%, 80%, and 100% of the LBNP protocol in 45°HUT (Panel A), supine (Panel B), and 45°HDT (Panel C) positions. Relative MCAv and PCAv during BL and at 20%, 40%, 60%, 80%, and 100% of the LBNP protocol in 45°HUT (Panel D), supine (Panel E), and 45°HDT (Panel F) positions. P_ET_CO_2_ during BL and at 20%, 40%, 60%, 80%, and 100% of the LBNP protocol in 45°HUT (Panel G), supine (Panel H), and 45°HDT (Panel I) positions. Mean data ± SEM is represented at each data point. No statistical comparisons between BL and percent of LBNP protocol are shown in the figure, these comparisons can be found in Table 2. Brackets between MCAv and PCAv data represents main effect (*P* < 0.05) between the two vessels. *P < 0.05, between baseline and percent of the LBNP protocol. **P < 0.05, between 100% of LBNP protocol and 20%, 40%, 60% of LBNP protocol. ***P < 0.05, between 100% of LBNP protocol and 40%, and 60% of LBNP protocol. NSD, no significant differences detected; HUT, head‐up tilt; LBNP, lower body negative pressure; PCA, posterior cerebral artery.

Table 2 and Figure 2 illustrate the cerebrovascular and respiratory responses during LBNP in each body position for all participants. MCAv was lower during LBNP compared to baseline in all three body positions (*P* < 0.001), however, there was no difference in MCAv during LBNP between body positions (main effect: *P* = 0.077). Similarly, during all LBNP stages in the supine position, PCAv was lower compared to baseline (*P* < 0.05). PCAv was lower compared to baseline during 45°HDT and LBNP (*P* < 0.05), with the exception of the 20% level of LBNP where it was the same as baseline (*P* = 0.095). In the 45°HUT position, PCAv was lower compared to baseline only during 60% (*P* = 0.049), 80% (*P* < 0.001), and 100% (*P* < 0.001) of maximal LBNP; at 100% of LBNP in the 45°HUT position, PCAv was lower compared to 45°HDT (*P* = 0.037). There was no difference in PCAv during LBNP between body positions (main effect: *P* = 0.583). MCA CVR and PCA CVR were higher during LBNP compared to baseline in each body position (*P* < 0.001). Additionally, MCA CVR was greater in 45°HUT compared to supine (main effect: *P* = 0.040).

### Anterior and posterior cerebral circulation responses during LBNP

MCAv and PCAv at baseline and during LBNP for each body position are illustrated in Figure 2. As expected, MCAv was greater than PCAv during LBNP in each body position (main effect, *P* < 0.001 for each body position; Fig. 2 panels A–C). In 45°HUT, MCAv was lower at 100% of LBNP protocol, compared to 20%, 40%, 60%, and 80% of LBNP protocol. In addition, there was a main effect for percent of LBNP protocol across the two vessels (*P* < 0.001), and there was an interaction effect for the cerebral vessel (MCAv and PCAv) and percent of LBNP (*P* < 0.001). In contrast, in the 45°HUT, PCAv was lower at 100% of LBNP protocol compared to 20% (*P* = 0.012), 40% (*P* = 0.012), and 60% (*P* = 0.030) of LBNP protocol. In both supine and 45°HDT positions, no differences were found during LBNP for both MCAv and PCAv. When normalizing CBV to baseline values during LBNP (i.e., percent change from baseline), MCAv and PCAv decreased from baseline throughout LBNP in each body position (*P* < 0.001), and there was no difference in relative changes from baseline during LBNP between the MCA and PCA in 45°HUT (main effect: *P* = 0.100), supine (main effect: *P* = 0.529), or 45°HDT (main effect: *P* = 0.407) (Fig. 2, panels D–F). Additionally, the relative decrease in CBV in the MCA and PCA was greater at 100% LBNP compared to 20%, 40%, 60%, and 80% LBNP in 45°HUT (*P* < 0.001), and compared to 40% and 60% LBNP in supine (*P* < 0.01).

**Figure 3:**
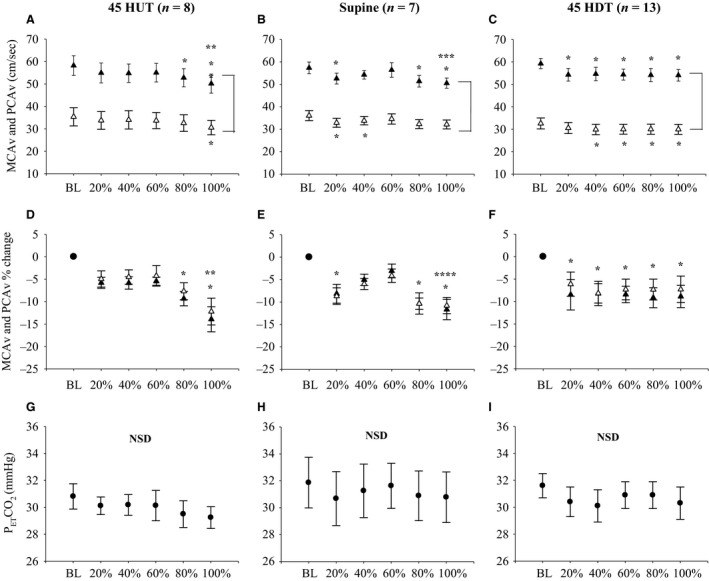
Absolute MCAv and PCAv responses to LBNP in each body position when P_ET_CO_2_ was appropriately controlled. (▲) represents MCAv data, (∆) represents PCAv data, (•) represents P_ET_CO_2_. Absolute MCAv and PCAv during baseline (BL) and at 20%, 40%, 60%, 80%, and 100% of LBNP protocol in 45°HUT (*n* = 8; Panel A), supine (*n* = 7; Panel B), and 45°HDT (*n* = 13; Panel C). Relative MCAv and PCAv during BL and at 20%, 40%, 60%, 80%, and 100% of LBNP protocol in 45°HUT (*n* = 8; Panel D), supine (*n* = 7; Panel E), and 45°HDT (*n* = 13; Panel F). P_ET_CO_2_ during BL and at 20%, 40%, 60%, 80%, and 100% of LBNP protocol in 45°HUT (*n* = 8; Panel G), supine (*n* = 7; Panel H), and 45° HDT (*n* = 13; Panel I). Mean data ± SEM is represented at each data point. Brackets between MCAv and PCAv data represent main effect (*P* < 0.05) between the two vessels. *P < 0.05, between baseline and percent of LBNP protocol. **P < 0.05, between 100% of LBNP protocol and 20%, 40%, 60% of LBNP protocol. ***P < 0.05, between 100% of LBNP protocol and 40%, and 60% of LBNP protocol. ****P < 0.05, between 100% of LBNP protocol and 60% of LBNP protocol. NSD, no significant differences detected; LBNP, lower body negative pressure; PCA, posterior cerebral artery.

We attempted to control P_ET_CO_2_ to baseline levels by coaching the participants’ ventilation during LBNP (see Fig. 2, panels G–I). In the 45°HDT position, there was no difference in P_ET_CO_2_ throughout LBNP compared with baseline among all participants (*n* = 13). In contrast, P_ET_CO_2_ was slightly lower compared to baseline in the supine posture at 20% (*P* = 0.023) and 100% (*P* = 0.004) of the LBNP protocol, and during 60% (*P* = 0.045), 80% (*P* < 0.001), and 100% (*P* < 0.001) of LBNP protocol in 45°HUT. Since we were unsuccessful at controlling P_ET_CO_2_ in 45°HUT and supine position, we analyzed data from a subset of participants where P_ET_CO_2_ was successfully controlled in 45°HUT (*n* = 8; *P* = 0.063) and supine positions (*n* = 7; *P* = 0.101) (see Fig. 3). As previously described, we were successful at controlling P_ET_CO_2_ in 45°HDT using all participants (*n* = 13).

### Anterior and posterior cerebral circulation responses during LBNP with no change in P_ET_CO_2_

Figure 3 illustrates the cerebrovascular responses to LBNP in participants who did not have a significant change in P_ET_CO_2_ during the LBNP protocol. The data in Figure 3 for 45°HDT (panels C, F, and I) are the same as in Figure 2 (*n* = 13). In the 45°HUT position, MCAv was significantly lower compared to baseline at 80% (*P* = 0.002) and 100% (*P* < 0.001) LBNP. Also, MCAv during 100% LBNP was lower compared to 20% (*P* = 0.007), 40% (*P* = 0.009), and 60% (*P* = 0.005) LBNP. In the supine position, MCAv was lower compared to baseline at 20% (*P* < 0.001), 80% (*P* = 0.008), and 100% (*P* ≤ 0.001) LBNP, and was lower during 100% LBNP compared to 40% (*P* = 0.024) and 60% (*P* = 0.001) LBNP. In 45°HUT, PCAv was only different between baseline and 100% LBNP (*P* = 0.006), while in the supine position, PCAv was different between baseline and 20% (*P* = 0.042), and 100% (*P* = 0.025) LBNP. When normalizing CBV to baseline values during LBNP (i.e., % change from baseline), we found that CBV in both the MCA and PCA was lower during 80% (*P* = 0.013) and 100% (*P* < 0.001) LBNP compared to baseline, and lower during 100% LBNP compared to 20% (*P* = 0.031), 40% (*P* = 0.025), and 60% (*P* = 0.018) in 45°HUT. In the supine position, relative CBV in both the MCA and PCA was lower during 20% (*P* = 0.011), 80% (*P* = 0.001), and 100% (*P* < 0.001) LBNP compared to baseline, and lower during 100% LBNP compared to 60% (*P* = 0.037).

When controlling for changes in P_ET_CO_2_, no differences were found between the relative reductions in MCAv and PCAv during LBNP in 45°HUT (*P* = 0.348), supine (*P* = 0.694), and 45°HDT (*P* = 0.407) (Fig. 3, panels D–F), consistent with the responses in the uncontrolled condition (Fig. 2, panels D–F).

## Discussion

The primary purpose of this study was to investigate the regional cerebrovascular responses to simultaneous body position and LBNP stress. We previously demonstrated that steady‐state tilt (HUT and HDT) has no effect on cerebrovascular reactivity to PaCO_2_ (Tymko et al. 2015). In addition, recent studies explored regional differences between the anterior and posterior circulations during LBNP (Deegan et al. 2010; Kay and Rickards 2016; Lewis et al. 2014, 2015; Ogoh et al. 2015), but there are no studies that have investigated regional CBF responses during LBNP and steady‐state tilt, both of which alter central blood volume and intracranial pressure. Our main findings were that (a) steady‐state tilt (i.e., 45°HUT, supine, and 45°HDT) had no effect on the CBV responses during LBNP in both the MCA and PCA, and (b) despite controlling for P_ET_CO_2_, both the MCA and PCA CBV decreased during LBNP in each body position, and the observed decrease in CBV for the MCA and PCA was the same between 45°HUT, supine, and 45°HDT.

### The physiological effects of steady‐state tilt

A change in body position has a profound effect on cardiovascular physiology. When moving from supine into the upright position (e.g., HUT), gravity draws blood down into the lower extremities causing a decrease in central blood volume and intracranial pressure; the opposite occurs for the HDT position (Bundgaard‐Nielsen et al. 2009; Macias et al. 2015; Murrell et al. 2011; Rosner and Coley 1986; Schwarz et al. 2002). In order to compensate for changes in body position, baroreceptors (e.g., aortic arch and carotid sinus) detect changes in MAP, and alter HR and vascular resistance in order to maintain CO, MAP, and thus, cerebral perfusion pressure (Cooke et al. 2004; Wehrwein and Joyner 2013).

Between the three body positions tested in this study (45°HUT, supine, and 45°DT), CO was similar, but the components that contribute to CO (HR and SV) were different between body positions. In the 45°HUT position, where a large opposing hydrostatic pressure gradient was present due to the effects of gravity, venous return was reduced resulting in a decrease in SV. In order to maintain CO and MAP, HR consequently increased (refer to Table 1). At baseline, there were no differences in MAP between supine and 45°HUT, but as expected, MAP increased with 45°HDT compared to supine, likely as a result of increased thoracic blood volume (Tymko et al. 2015). Interestingly, the HDT‐associated increases in thoracic blood volume was not reflected in changes in SV or CO at baseline, meaning that the observed increase in MAP was likely due to increases in peripheral resistance.

We attempted to control P_ET_CO_2_ by coaching participants’ breathing pattern throughout LBNP to baseline levels, as changes in P_ET_CO_2_ (reflective of changes in PaCO_2_) can alter the resistance of downstream cerebral arterioles, drastically changing CBV (Kety and Schmidt 1948; Wolff et al. 1930). Fortunately, P_ET_CO_2_ did not change between body positions at baseline, so any body position related changes in MAP appear to be countered with changes in intracranial pressure (Tymko et al. 2015).

### Cerebrovascular responses to steady‐state tilt and LBNP

There is limited literature on the effects of body position on CBF responses during LBNP (Deegan et al. 2010). In a previous study, no differences were reported between CBV in the MCA and volumetric blood flow in the vertebral artery, which is located proximal to the PCA, during combined HUT and LBNP, but no responses were measured during HDT (Deegan et al. 2010). Another study compared regional differences in CBF by measuring volumetric flow within the internal carotid and vertebral arteries during thigh cuff release in supine and HUT positions (Sato et al. 2012a). Interestingly, these investigators report that body position (supine vs. HUT) elicited a differential CBF response, where internal carotid artery blood flow was reduced during HUT compared to supine. In contrast, when measuring CBV responses during steady‐state tilt and LBNP, we found that body position had no effect on MCAv and PCAv during LBNP in HUT, or during the relatively unexplored body position of HDT. This discrepancy with previous work could be due to several reasons. First, we measured regional cerebral blood velocity using TCD rather than volumetric flow, opposite to the previous report that used thigh cuff release (Sato et al. 2012a). As such, it is possible that there are differences in cerebral blood flow regulatory mechanisms between intra‐ and extracranial arteries, and between the anterior and posterior intracranial circulations. The validity of using TCD for assessing flow responses has been debated, as CBV is only indicative of CBF if the diameter remains unchanged within the insonated conduit cerebral arteries (i.e., MCA and PCA; see further discussion below) (Aaslid et al. 1982; Giller 2003). Second, another potential reason for the observed differences between experimental studies is that the stimulus for central hypovolemia was different; thigh cuff release combined with HUT (Sato et al. 2012a) versus LBNP and HUT (Deegan et al. 2010). Thigh cuff release results in a rapid, transient drop in central blood volume and MAP (i.e., within 1 min), while LBNP progressively decreases central blood volume, which results in little to no change in MAP until presyncope is reached. This means that potentially, central hypovolemia in‐and‐of itself does not elicit body positional related changes in CBF, and that a decrease in MAP (i.e., hypotension) is what governs the observed regional differences in cerebral blood flow (Sato et al. 2012a). In the 45°HUT position, we observed a decrease in MAP at 100% LBNP, probably due to the high volume of participants that reached presyncope. However, no differences between MCAv and PCAv were observed at this time period, and this is likely because the observed hypotension was not to the same extent that is typically seen with thigh cuff release (i.e., transient drop in MAP by ~15–20 mmHg). To date, there is only one study that has measured regional cerebral blood flow responses to LBNP until presyncope, while controlling for PaCO_2_ (Lewis et al. 2015). It was found that at the point of presyncope (characterized by hypotension), the posterior cerebral circulation (vertebral artery) was better at maintaining blood flow compared to the anterior cerebral circulation (internal carotid artery). Our results contrast these previous findings, however, the aim of our experiment was not to reach presyncope in each body position, and it is possible that the degree of hypotension reached in our study during LBNP (particularly in HUT) was not significant enough to elicit a differential regional cerebrovascular response.

### Regional cerebral blood velocity during steady‐state tilt and LBNP

Historically, when investigating the cerebrovascular response to specific stimuli, the MCA was the vessel of interest, primarily because it is relatively easy to insonate with TCD, and it supplies a large region of the brain with blood and nutrients, so it is a sound “representation” of CBF (Schoning et al. 1994). However, recent evidence suggests that the anterior and posterior cerebral circulations respond differently to physiological stimuli, notably to changes in PaCO_2_ (Sato et al. 2012b; Skow et al. 2013; Willie et al. 2012) and to LBNP (Kay and Rickards 2016; Lewis et al. 2015; Ogoh et al. 2015). In response to LBNP, recent work suggests that blood flow within the posterior cerebral circulation is better preserved compared to the anterior cerebral circulation (Kay and Rickards 2016; Ogoh et al. 2015). In contrast, it has also been found that the posterior cerebral artery is more reactive to hypotension compared to the anterior cerebral artery (Lewis et al. 2015).

Previous studies demonstrated that CBF declines with LBNP, but unfortunately, PaCO_2_ is rarely controlled during these studies and is often overlooked as a potent vasoconstrictor stimulus under these conditions (Brown et al. 2003; Levine et al. 1994; Ogoh et al. 2015; Rickards 2015). The advantage of this study is that we assessed the cerebrovascular responses to LBNP while simultaneously controlling for P_ET_CO_2_ (a surrogate for PaCO_2_; see Fig. 3). Our data show that CBV in the MCA and PCA still decrease from baseline during LBNP despite controlling P_ET_CO_2_, in fact, CBV responds similarly to LBNP between our overall mean data (Fig. 2) and P_ET_CO_2_‐controlled data (Fig. 3). Although we did not control P_ET_CO_2_ statistically in all of our participants (*n* = 13), our data suggest that the small decreases in P_ET_CO_2_ observed during LBNP (~2 mmHg in supine, ~3 mmHg in HUT) was not great enough to elicit a significant differential response between the two groups (i.e., Fig. 2 vs. 3). In addition, there was no difference in the relative decrease in CBV between the MCA and PCA, supporting the conclusion that velocity in the intracranial cerebral vessels respond in a similar fashion to submaximal LBNP. Previous literature has shown that the anterior and posterior cerebral circulations respond similarly during LBNP stress (Deegan et al. 2010; Lewis et al. 2014), while others have shown that there might be regional differences in CBF responses to LBNP (Kay and Rickards 2016; Lewis et al. 2015; Ogoh et al. 2015) and thigh cuff release (Sato et al. 2012a). Interestingly, these studies used both TCD and duplex Doppler ultrasound measurement techniques, suggesting that the discrepancy may be due to other differences in methodology, such as the magnitude of central hypovolemic stress (maximal vs. submaximal). Nevertheless, our data add to the growing literature exploring regional CBF responses during central hypovolemia, and add a novel component by highlighting CBV measurements in both HUT and HDT positions.

### Methodological considerations

Despite providing valuable mechanistic insight, our study had several limitations. The stimulus of central hypovolemia induced by LBNP was different between body positions, as illustrated by the differential cardiovascular responses to LBNP between HDT, supine, and HUT (see Table 1). However, in each body position, a significant increase in heart rate and reduction in stroke volume occurred during LBNP, indicating that central blood volume was decreasing in each of the three body positions, in fact, one participant was excluded from data analysis for being extremely LBNP intolerant, and reached presyncope in HDT. However, central blood volume was not directly measured, so the difference in LBNP stimulus between the body positions cannot be accurately quantified. In addition to central blood volume not being directly measured, intracranial pressure was also not measured. However, it is known that intracranial pressure is altered during changes in body position (Macias et al. 2015; Rosner and Coley 1986; Schwarz et al. 2002). For example, previous reports suggest that for every 10 degrees of HUT, intracranial pressure decreases by 1 mmHg (Rosner and Coley 1986). Additionally, LBNP may mildly reduce intracranial pressure (Macias et al. 2015), but this is based on intraocular pressure, a surrogate for intracranial pressure. To date, no studies have explored the effects of LBNP on intracranial pressure measured directly across the skull.

As previously mentioned, in terms of measuring CBF, we assessed CBV via TCD as a surrogate for CBF, with the assumption that the cross‐sectional area of the insonated vessel does not change. Despite previous demonstrations that MCA diameter does not change during alterations in PaCO_2_ and mild LBNP challenges (Serrador et al. 2000), there is still debate. More recent studies suggest that hypo‐ and hypercapnia may in fact elicit changes in MCA diameter (Ainslie and Hoiland 2014; Coverdale et al. 2014; Verbree et al. 2014). At present, measures of extracranial arteries (e.g., internal carotid and vertebral arteries) can provide the most reliable noninvasive measures of global cerebral volumetric inflow (Hoiland et al. 2015; Willie et al. 2012). However, it would have been very difficult to perform these types of measurements due to the positioning of our participants. Participants were suspended within the tilt‐LBNP apparatus, approximately, a meter above the ground, which would make it difficult getting reliable measurements, as ultrasound measures of the internal carotid and vertebral cerebral arteries would require the sonographer to consistently insonate the vessel at the same angle, in each body position.

Lastly, although mean P_ET_CO_2_ changed only very little from baseline during LBNP (~2 mmHg in supine, and ~3 mmHg in HUT), we were unable to successfully coach P_ET_CO_2_ levels to remain at baseline values in several of our participants during LBNP in the 45°HUT and supine position due to the participants hyperventilating during LBNP. To investigate the effects of LBNP on regional CBV independent of changes in PaCO_2_, we performed analysis on a subset of participants (45°HUT, *n* = 8; supine, *n* = 7) that had no statistical change in P_ET_CO_2_ during the LBNP protocol. Although the number of participants included in analysis decreased, we still achieved adequate statistical power (>0.80) for all CBV comparisons, however, we lacked statistical power for P_ET_CO_2_ in 45°HUT (0.413), and supine (0.313), due to the smaller sample size (Fig. 3). Despite this, we demonstrated that the CBV responses to LBNP were similar regardless of PaCO_2_ control. A future approach could be to include end‐tidal gas control using an end‐tidal forcing system.

### Perspectives and significance

We measured the regional cerebrovascular responses to −50 mmHg of steady‐state LBNP in three different body positions, which were used as modalities to alter central blood volume and intracranial pressure. We found that (a) body position had no effect on CBV responses during LBNP within both the MCA and PCA, and (b) despite controlling for P_ET_CO_2_, both the MCA and PCA CBV decreased during LBNP in all body positions, and (c) the observed decrease in MCAv and PCAv was the same in 45°HUT, supine, and 45°HDT. Our novel tilt‐table LBNP apparatus allowed us to conduct the first study that measured regional CBV during LBNP in both HUT and HDT positions. Our results demonstrate that body positional changes in central blood volume and intracranial pressure were not substantial enough to alter CBV responses to LBNP, illustrating the regulatory capacity regulatory capacity of the brain. Moreover, in contrast to some previous literature, we found that there are no regional differences in cerebral blood flow between anterior and posterior cerebral circulations during central hypovolemia, despite the differences in brain regions (e.g., frontal lobe vs. brainstem) to which the anterior and posterior cerebral circulations provide oxygen and nutrients.

## Conflict of Interest

None declared.
